# Alginate/Gelatin Hydrogels Reinforced with TiO_2_ and β-TCP Fabricated by Microextrusion-based Printing for Tissue Regeneration

**DOI:** 10.3390/polym11030457

**Published:** 2019-03-11

**Authors:** Rodrigo Urruela-Barrios, Erick Ramírez-Cedillo, A. Díaz de León, Alejandro J. Alvarez, Wendy Ortega-Lara

**Affiliations:** 1 Tecnologico de Monterrey, Escuela de Ingeniería y Ciencias, Av. Eugenio Garza Sada #2501 Sur, Monterrey, NL 64849, Mexico; A00805049@itesm.mx (R.U.-B.); a00806274@itesm.mx (E.R.-C.); adiazdeleon@tec.mx (A.D.d.L.); 2 3D FACTORY MX, Ramón Treviño #1109 Col. Terminal, Monterrey, NL 64580, Mexico; 3 Laboratorio Nacional de Manufactura Aditiva y Digital (MADIT), Autopista al Aeropuerto, Km., 9.5, Calle Alianza Norte #100, Parque PIIT, Apodaca, NL 66629, Mexico

**Keywords:** hydrogel, 3D printing, alginate, gelatin, tissue engineering

## Abstract

Three-dimensional (3D) printing technologies have become an attractive manufacturing process to fabricate scaffolds in tissue engineering. Recent research has focused on the fabrication of alginate complex shaped structures that closely mimic biological organs or tissues. Alginates can be effectively manufactured into porous three-dimensional networks for tissue engineering applications. However, the structure, mechanical properties, and shape fidelity of 3D-printed alginate hydrogels used for preparing tissue-engineered scaffolds is difficult to control. In this work, the use of alginate/gelatin hydrogels reinforced with TiO_2_ and β-tricalcium phosphate was studied to tailor the mechanical properties of 3D-printed hydrogels. The hydrogels reinforced with TiO_2_ and β-TCP showed enhanced mechanical properties up to 20 MPa of elastic modulus. Furthermore, the pores of the crosslinked printed structures were measured with an average pore size of 200 μm. Additionally, it was found that as more layers of the design were printed, there was an increase of the line width of the bottom layers due to its viscous deformation. Shrinkage of the design when the hydrogel is crosslinked and freeze dried was also measured and found to be up to 27% from the printed design. Overall, the proposed approach enabled fabrication of 3D-printed alginate scaffolds with adequate physical properties for tissue engineering applications.

## 1. Introduction

Three-dimensional (3D) printing technologies have become an attractive manufacturing process to fabricate 3D scaffolds in tissue engineering. Recent research has focused on the fabrication of complex-shaped structures that closely mimic biological organs or tissues. It is expected that 3D-printed constructs will be able to replace as well as regenerate defective tissues or injured functional tissues and organs. An interesting and challenging application of tissue engineering is the repair of oral and craniofacial bone defects [1,2,3]. Different types of biomaterials have emerged to treat these tissues in the form of injectable and non-injectable scaffolds. An ideal scaffold must include three components: a scaffolding matrix fabricated with biodegradable materials, cells, and signaling molecules [4]. Hydrogels scaffolds have been revolutionizing the fields of material sciences, biomedical engineering, and the pharmaceutical industry [5]. They exhibit unique properties, including biocompatibility, flexible synthesis methods, degradability, and a wide range of possible constituents [6]. Research is intensely focused on the mechanical properties of the hydrogels, while maintaining their biocompatibility [7,8,9].

Alginate hydrogels belong to a family of linear copolymers capable of forming stable gels in the presence of calcium or other divalent cations [10,11]. Alginates can be effectively manufactured into porous three-dimensional biodegradable networks, and they have been used in combination with other components in tissue engineering applications [12,13,14,15,16,17,18]. Several authors have recently reported the use of alginate combined with other materials in the tissue engineering field, achieving defined microarchitectures with the help of additive manufacturing [16,19,20,21]. As a reinforcing agent, calcium phosphates, such as tricalcium phosphate (β-TCP), have been used for bone regeneration due their osteoconductive properties by stimulating the cell proliferation and the production of growth factors [22]. Another example is titanium dioxide (TiO_2_), which has been used in several applications for its properties, which include bioactivity, self-cleaning glass, and being a semiconductor. It has been demonstrated that the addition of titanium dioxide to the hydrogels can improve bone tissue regeneration [23,24,25]. Shirai et al. confirmed the antimicrobial activity of TiO_2_ against periodontal pathogen after UV irradiation due to the photocatalytic effect, where free radicals induce damage in the bacteria cell wall [26]. Both characteristics, bioactivity and antimicrobial activity, are highly desirable in tissue engineering. Some of the challenges with respect to alginates designed for additive manufacturing (3D printing) are the printability, accuracy, and shape fidelity of the hydrogel scaffolds. These problems are associated with the rheological properties of the alginate solution, where a weak structure of the hydrogel ink can compromise the mechanical properties, making it difficult for the hydrogel to retain a shape in a predesigned geometry [9,27,28,29].

In this work, alginate/gelatin hydrogels reinforced with titanium dioxide and β-tricalcium phosphate were fabricated by microextrusion-based printing. Hydrogels were characterized by Fourier transform infrared spectroscopy (FTIR), scanning electron microscopy (SEM), and X-ray diffraction (XRD). Mechanical and rheological properties of the hydrogels were also evaluated. The effect of the addition of TiO_2_, β-TCP, and gelatin on the mechanical properties of the alginate scaffolds was studied. The printability of hydrogels was related to the rheological properties of the pre-crosslinked alginate hydrogel. Three different printed shapes/geometries were fabricated. The shape fidelity of the printed scaffolds was also evaluated. The proposed approach is useful for fabricating 3D-printed alginate scaffolds with adequate physical properties that can be utilized in tissue engineering applications.

## 2. Materials and Methods

### 2.1. Materials and Methodology

#### 2.1.1. Materials 

Sodium alginate (CAS no. 9005-38-3), a polysaccharide consisting of mannuronic/guluronic acid (M/G) ratio of 34%/66%, Titanium dioxide (TiO_2_) nanopowder (21 nm, CAS no. 12188-41-9), β-Tricalcium phosphate (β-TCP) (CAS no. 7758-87-4, >95% pure), was purchased from Sigma Aldrich (Saint Louis, MO, USA, USA). Gelatin (CAS no. 9000-70-8) and Calcium chloride (CaCl_2_) (CAS no. 10043-52-4, >96% pure) was obtained from CTR Scientific (Control Técnico y Representaciones Scientific, Monterrey, Mexico). Precision tips SNS-D 051/025 were purchased from Nordson (Inner-0.25 mm) (Nordson Corporation, OH, USA). 

#### 2.1.2. Pre-Crosslinked Hydrogel Solution Preparation and Characterization 

First, deionized water was used to dissolve 0.20 *w*/*v* % CaCl_2_ to prepare pre-crosslinked hydrogels. Once the CaCl_2_ was completely dissolved with the aid of a magnetic stirrer, sodium alginate (2 *w*/*v* %) and gelatin were slowly added. The solution was stirred for at least two hours at 1000 rpm and maintaining a temperature of 55 °C. To avoid agglomeration, the TiO_2_ nanoparticles (0.1 *w*/*v* %) and the β-TCP (1.0 *w*/*v* %) were added before the biopolymers. In Table 1, the different hydrogel solutions are presented along with their individual formulations. 

#### 2.1.3. Rheology 

Once the different pre-crosslinked hydrogel solutions were ready, they were characterized using a plate rheometer Anton Paar MCR 301 (Anton Paar GmbH, Graz, Austria). A flow curve was obtained for each sample from 0.1 to 100 1/s shear rate to obtain the viscoelastic behavior of the hydrogel solution before cross-linking with different concentrations of calcium chloride. Flow curves were also obtained to study the effect of the composition of the hydrogel (gelatin, TiO_2_, and β-TCP) on viscosity. Strain tests were also performed on the hydrogel solutions at a constant angular frequency of 10/s to measure the storage and loss modulus of the samples. 

#### 2.1.4. Crosslinked Hydrogel Preparation 

To crosslink the hydrogels, a 6 *w*/*v* % CaCl_2_ solution was prepared. The pre-crosslinked hydrogels were immersed in the calcium chloride solution for at least 24 h to ensure complete crosslinking. After crosslinking, the hydrated hydrogels are washed with deionized water at least five times to remove all the excess calcium chloride left in the material. 

#### 2.1.5. X-ray Diffraction 

XRD techniques were used to validate the crystallinity of the nanoparticles inside the matrix of the composite hydrogel. XRD was recorded in the 2θ range between 10° and 85° and a step size of 0.026 using a PANalytical Empyrean diffractometer (PANalytical, Almelo, The Netherlands), CuKα radiation, of which the wavelength was λ = 1.5406 Å. The applied voltage was 45 kV and the current was 40 mA. Samples were pulverized before measuring.

#### 2.1.6. FTIR 

Functional groups resolution were identified of each hydrogel sample with an infrared spectrometer coupled with Fourier transform Perkin Elmer (Waltham, MA, USA) model Spectrum 400 recorded in the wavenumber range of 4000–400 cm^−1^ at room conditions.

#### 2.1.7. Pore Size Analysis and Microscopy 

For the pore size analysis, disc-shaped hydrogels were synthesized following the crosslinked hydrogel preparation described before. The discs were first frozen at −80 °C and then lyophilized in a LabConco FreeZone freeze dryer (LabConco, Kansas City, MO, USA) for 48 h at −49 °C, and 0.04 mbar. The resulting dried discs were sliced carefully using a razor and tweezers, and then the cross-section of the discs was analyzed in EVO MA25 Zeiss Scanning electron microscope (Zeiss, Jena, Germany) with an accelerating voltage of 20 kV and high vacuum. Three pictures were taken from each disc, ImageJ (National Institutes of Health, Bethesda, MD, USA) processing image program was utilized to calculate the average pore size. 

#### 2.1.8. Mechanical Tests 

The mechanical tests of the crosslinked hydrogel samples were characterized under the ASTM D638 standard Type V specimens for tensile tests. The pre-crosslinked solutions were poured into the molds and then they were crosslinked with the 6 *w*/*v* % CaCl_2_ solution. The resulting dumbbell shape hydrogel specimens were washed with deionized water and then left to dry for later testing in the Instron Universal Machine 3365 (Instron, Norwood, MA, USA). Samples were lyophilized for testing at 1 mm/min following the same norm for rigid and semirigid specimens. Four samples of each of the hydrogels were measured and the average elastic modulus was obtained. 

### 2.2. Design and Manufacturing Process

#### 2.2.1. Design Models 

**Figure 1:**
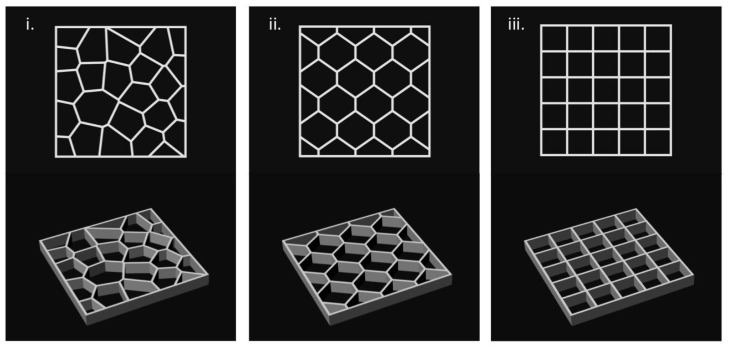
Scaffold designs: (**a**) voronoi, (**b**) hexagon, (**c**) grid.

Scaffolds were designed using Rhinoceros 5.0 (Mc Neel & Associates, WA, USA), a 3D modeling software with the grasshopper plugin. Three types of scaffolds were designed and are shown in Figure 1: grid, voronoi, and hexagons (20 mm × 20 mm × 2 mm). Rhinoceros used the grasshopper plugin, a graphical algorithm editor for the fabrication of parametric forms without the scripting experience [30]. The parameters for this module were selected in order to have reproducible and well-structured scaffolds. The parameters were line thickness (0.40–0.60 mm), cell size (depending on the cell number), and cell number (25–35).

#### 2.2.2. Manufacturing Process 

**Figure 2:**
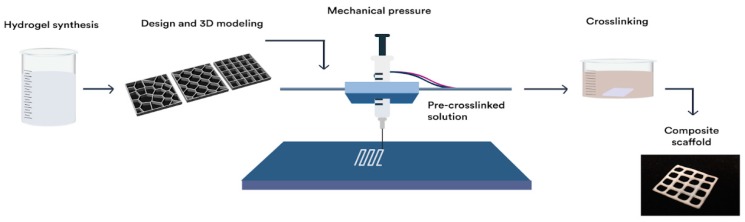
Illustration of printing process of the scaffolds. From synthesis of the solution, computer-aided design, extrusion computer-controlled, crosslinking of the scaffold and crosslinked printed design.

For the extrusion process of hydrogels, a RepRap Mendelmax 3D printer (Fundació CIM, Barcelona, Spain) with a work volume of 20 mm × 20 mm × 15 mm was modified. The extrusion process was modified to work with a piston extruder such as a 10 mL (14 mm of diameter) syringes (20 G, BD Ltd., Franklin Lakes, NJ, USA) with a needle of internal diameter of 0.2 mm (SNS-D 051/025, Nordson). For the generation of the G-code, the STL was introduced into an open source software Cura 15.04.6 (Ultimaker, Geldermalsen, The Netherlands). The parameters for the scaffold production were optimized. Scaffolds were fabricated with a layer height of 0.15 mm, a shell thickness of 0.25 mm, and continuous flow of 100%. For the extrusion process, a speed of 4.5 mm/s was used with a fill density of 100%. Change in temperature was not required in this process. Qualitative analysis of the printed structures was performed using a SteREO Discovery.V8 equipped with a CCD camera AxioCam HRc (Carl Zeiss Micro imaging GmbH, Jena, Germany) and AxioVision 4.8 software. To measure line deformation in scaffolds as the height of the design increases, the grid design was printed with height of 0.15, 0.75, and 1.5 mm (1, 5, and 10 layers respectively). Afterwards, four different line widths of the printed grid were measured with the SteREO and reported for each of the hydrogels (HG-1 to 6). Furthermore, for the reduction in the line width in the different processes, the same methodology was used for the grid designs after crosslinking and after freeze drying and a comparison was made. The whole manufacturing process is depicted in Figure 2. 

#### 2.2.3. Die Swell Behavior 

Also called the Barus effect, this was measured with a high-speed DataPhysics OCA 15EC system (DataPhysics Instruments GmbH, Filderstadt, Germany). Several images using the SCA20_U software (DataPhysics Instruments GmbH, Filderstadt, Germany) were taken while a drop of material was extruded. The width of the hydrogel solution drop was measured 0.08 mm from the end of the tip, which is the distance in the actual printing process from the end of the tip of the needle to the build surface of the machine. 

## 3. Results and Discussion

### 3.1. Study of the Rheological Properties of the Hydrogel Ink

The suitability of a hydrogel for a specific fabrication process mainly depends on its physicochemical properties under the conditions imparted by the specific fabrication instrument. The major physicochemical parameters that determine the printability of a hydrogel are its rheological properties [31]. Pre-crosslinked alginate inks were prepared by mixing sodium alginate (2 *w*/*v* %) with 0%, 0.05%, 0.10% and 0.15% calcium chloride solutions. The rheological properties of the hydrogel inks were characterized using a plate rheometer.

**Figure 3:**
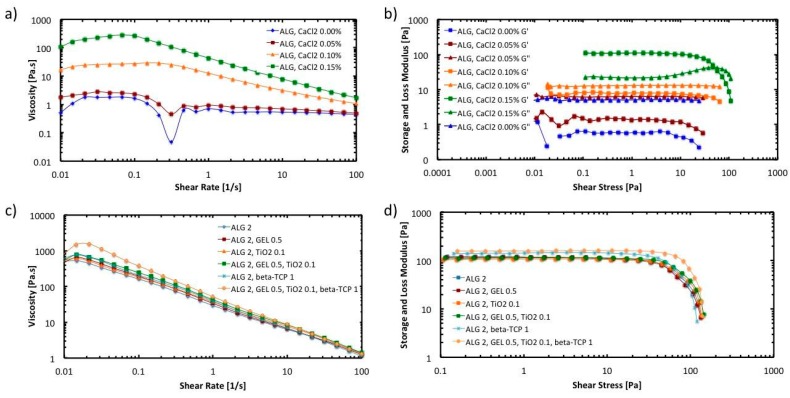
Rheology tests: (**a**) Flow curve for pre-crosslinked hydrogel inks; (**b**) Amplitude sweep for pre-crosslinked hydrogel inks; (**c**) Flow curve of hydrogels with different compositions with 0.20% of pre-crosslinker; (**d**) Amplitude sweep (Storage modulus) of hydrogels with different compositions with 0.20% of pre-crosslinker.

Figure 3 shows the results of the rheological characterization of the hydrogel inks. In Figure 3a, the flow behavior under shear is presented. The viscosity of the hydrogel inks is plotted as a function of the shear rate. It was found that altering the concentration of calcium chloride resulted in a practical method of controlling the viscosity of the alginate ink. As the concentration of calcium chloride gets higher, the viscosity of the hydrogel increases due to a higher percentage of crosslinked alginate molecules ionically bonded with calcium. 

It can also be observed that hydrogel ink exhibits a shear-thinning behavior or pseudo-plasticity, which is characterized by decreasing viscosity with increasing shear rate. This behavior is caused by shear-induced reorganization of the polymer chains to a more stretched conformation, which leads to decreased entanglement and, therefore, viscosity [32]. As the concentrations of calcium chloride increases, the relative reduction in viscosity induced by shear become greater. At a shear rate of 100 s^−1^ the viscosity of the hydrogel ink prepared with 0.15% calcium chloride is approximately an order of magnitude lower than the value at 0.1 s^−1^.

It has been reported that the viscosity of the hydrogel ink directly influences shape fidelity after deposition [33]. Printing fidelity generally increases with increasing viscosity [34]. As has been noted by other researchers [31], the pseudo-plasticity phenomena observed in the hydrogel ink implies a decreased shear stress at the high shear rates that are present inside a syringe orifice during fabrication, followed by a sharp increase in viscosity resulting in a high printing fidelity upon deposition.

To have a better insight of the viscoelastic properties of the hydrogel inks, oscillatory strain amplitude sweep measurements were conducted at a constant angular frequency of 10/s. As can be observed in Figure 3b, the storage modulus (G′) is higher than the loss modulus (G″) in the linear viscoelastic region when the calcium chloride concentration is 0.15 *w*/*v* %. This indicates that the hydrogel ink shows a gel-like or solid structure and can be termed a viscoelastic solid material. On the other hand, G″ is higher than G′ in the linear viscoelastic region when the calcium chloride concentration is lower than 0.15%. This indicates that hydrogel inks display a fluid structure and can be termed a viscoelastic liquid. For 3D printing applications, it is essential that the ink behave as a viscoelastic solid material to avoid the collapse of the scaffold structure when it is being printed.

Further tests were conducted to evaluate the effect of the addition of gelatin, TiO_2_ nanoparticles, and β-TCP on the rheological properties of the hydrogel ink. The assessment included six different hydrogel inks (HG-1 to HG-6). The composition of the inks is described in Table 1. Figure 3c,d shows the results of the viscosity curve and the strain test, respectively. It can be observed that the addition of gelatin or TiO_2_ nanoparticles, seem to not affect the viscosity of the solution nor their viscoelastic behavior. Nevertheless, addition of β-TCP (1.0 *w*/*v* %) together with gelatin and TiO_2_ (HG-6) increases the viscosity of the pre-crosslinked hydrogel ink in the low shear rate range. The relative reduction in viscosity induced by shear become greater in HG-6. Also, the size of the linear viscoelastic region in HG-6 is larger than the rest of the inks. This region indicates the range in which the shear stress can be applied without destroying the structure of the hydrogel [35]. Overall, the rheological properties of the hydrogel inks are favorable for the extrusion process in the 3D printer [31]. In the syringe, the hydrogel chains form a temporary network with high viscosity. Upon disposing through a needle, the network is broken up by shear and the polymer chains align, reducing the viscosity by orders of magnitude. Directly after removal of shear stress, the network is restored, resulting in a high printing fidelity upon deposition.

### 3.2. FTIR and XRD Characterizations

**Figure 4:**
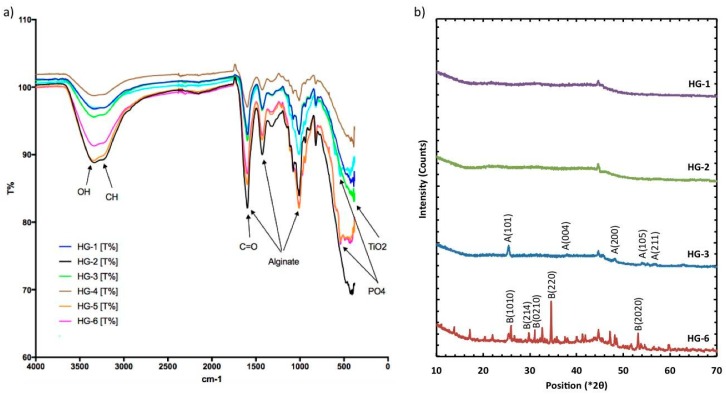
(**a**) FT-IR Identification of functional groups and major components of the dried composite hydrogels. Refer to Table 1 for the hydrogel compositions. (**b**) XRD plots of dry hydrogel where TiO_2_ (A) is identified as the crystal nanoparticles and calcium phosphate (B) as the TCP component.

Comparing FTIR spectra (Figure 4a) of the different hydrogels, it was observed that the alginate chemical structure was maintained with the addition of other components. The bands at 3273 cm^−1^ represent the stretching vibrations of O-H in calcium alginate, while the peaks at 1590 and 1410 cm^−1^ represent the stretching vibrations of C=O, and finally, the strong peak observed at 1030 cm^−1^ is assigned to COC and C-C vibrations. Other bond vibrations, such as the one of Ti-O, could also be identified in the figure for the hydrogels where this component was added, such as the HG-3, HG-4, and HG-6 hydrogels. The sharp peaks representing the stretching of PO_4_ in β-TCP are at 561 and 607 cm^−1^. In Figure 4b, the results of the XRD test with peak identification can be found. Alginate and gelatin showed no characteristic peaks, given that they form an amorphous polymer. The XRD results showed the crystallized form of TiO_2_ nanopowder. The crystalline form of TiO_2_ is anatase, with characteristic peaks at 21°, 23°, 48° (JCPDS 01-073-1764), which can be found in the samples where the oxide was added (HG-3 and HG-6). Of the hydrogels containing β-TCP, HG-6 is shown in Figure 4b, showing characteristics peaks of calcium phosphate at 23°, 31°, 35°, and 53° (JCPDS 01-073-4879).

Several studies have been performed to characterize the compatibility between alginate hydrogels and TiO_2_, gelatin, and β-TCP. Dong et al. reported strong evidence of the intermolecular interactions and good molecular compatibility between alginate and gelatin. Using FTIR analysis, they found that the absorption band concerned the stretching vibration of N-H group bonded to O-H group shifted to a lower wavenumber, suggesting an increase in the hydrogen bonding [36]. Zhao et al. studied the compatibility between calcium alginate and TiO_2_ using Fourier transform infrared spectrometry. They reported that TiO_2_ particles had good compatibility with calcium alginate, because there were many hydroxyl groups on the surface of TiO_2_, which formed hydrogen bonds with the hydroxyl groups in the alginate hydrogel [37]. Das et al. proposed a mechanism to explain the attachment of TCP ceramic particles in the alginate network. In the presence of CaCl_2_, the –COO^−^ groups of alginate crosslink with Ca^2+^ ions through electrostatic interactions and form ionic crosslinked alginate-TCP gel. Alginate moieties connect with each other through a cooperative mechanism and form an “egg-box” pattern. Basically, the divalent cations (Ca^2+^) connect alginate chains via ionic interaction and make interconnected layers around the eggs [38]. In this work, the possible interaction between TiO_2_, gelatin, β-TCP, and the hydrogel matrix was identified by FTIR analysis. It is apparent that physical interaction, mainly H-bonding, is responsible for binding the components into the alginate hydrogel. HG-1 is the alginate hydrogel without components, whereas HG-6 is the alginate hydrogel with TiO_2_, gelatin, and β-TCP. In the FTIR spectrum of HG-1, the band at 3273 cm^−1^ represents the stretching vibrations of O-H in calcium alginate. In the FTIR spectrum of HG-6, the peak value for O-H stretching appeared at 3233 cm^−1^. The shifting of peaks of –OH group to a lower wavenumber indicates physical interaction (H-bonding) and good compatibility between the components of the composite hydrogel. 

### 3.3. Porosity and Structure

**Figure 5:**
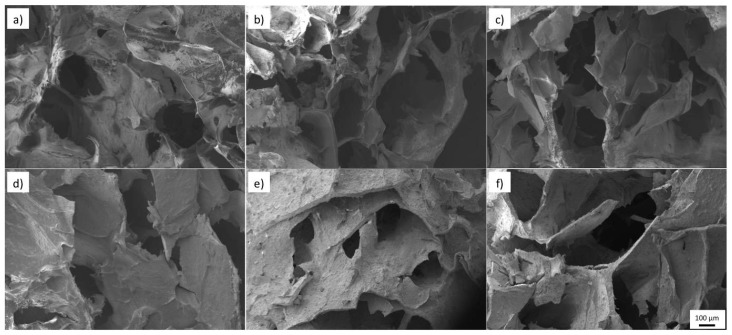
SEM morphology of hydrogels (**a**) *HG-1* (**b**) *HG-2* (**c**) *HG-3* (**d**) *HG-4* (**e**) *HG-5* (**f**) *HG-6.*

**Figure 6:**
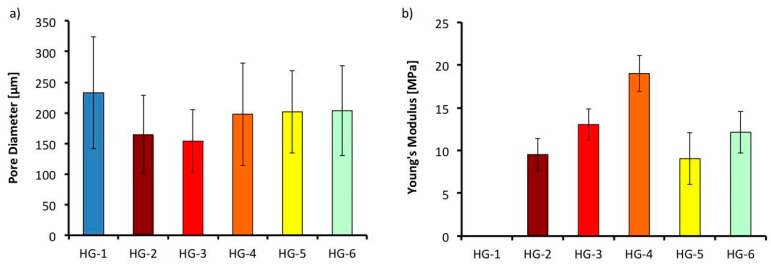
(**a**) Average pore size of the different hydrogels obtained from SEM images (**b**) Young’s Modulus of the different hydrogels obtained from tensile test in the Universal machine.

All the hydrogels in Figure 5 show high porosity, a property sought after in tissue engineering for biological delivery. An average pore size that ranges from 150 to 240 μm is observed in Figure 6a. A trend can be observed with the average pore size of the samples. When a second biopolymer, in this case, gelatin in HG-2, or TiO_2_ nanopowder in HG-3, is added to the solution, a reduction of the average pore size is observed. This reduction can be related to the increased degree of entanglement of the alginate polymer chains and the change in the hydrogels internal structure, both caused by the addition of a second component. Nevertheless, its average pore size and standard deviation (150–450 μm) is in the range of an adequate scaffold pore size to promote cell proliferation of bones and cartilages [8].

### 3.4. Mechanical Test

To determine the potential use of the alginate/gelatin composite hydrogel in tissue engineering applications, mechanical properties were measured with tensile tests of the dried hydrogels. McKee et al. measured different values of elastic modulus of soft tissue engineering that ranges all the way from 2 MPa for spinal cords, to 560 MPa in some tendons with tensile tests [39], but for oral soft tissues, Goktas et al. showed that gingival tissue shows an elastic modulus of 19.7–56.2 MPa [40]. Results of the tensile test of the different hydrogels can be found in Figure 6b. The elastic modulus obtained from the different hydrogel composites ranges from 9–20 MPa. The reference hydrogel HG-1, containing alginate alone, was too fragile even to measure, breaking up while handling the probe. Nevertheless, as gelatin (HG-2) or the TiO_2_ nanoparticles (HG-3) are added, the elastic modulus increases to 9 MPa and to 13 MPa respectively. Furthermore, when both components (gelatin and nanoparticles in HG-4) are added, its elastic modulus is further increased to 20 MPa. This is believed to happen due to the increased entanglement of the crosslinked polymer chains when a second component is added—the same behavior that caused the reduction in the average pore size discussed previously. When β-TCP is added to the hydrogel probe, the elastic modulus decreases. Instead of promoting entanglement of the alginate chains, β-TCP causes an earlier fracture of the hydrogel probes. This is thought to be caused by the high percentage of calcium phosphate added, which is in the ratio of 1:2 of alginate in the solution. 

### 3.5. Die Swell Behavior

**Figure 7:**
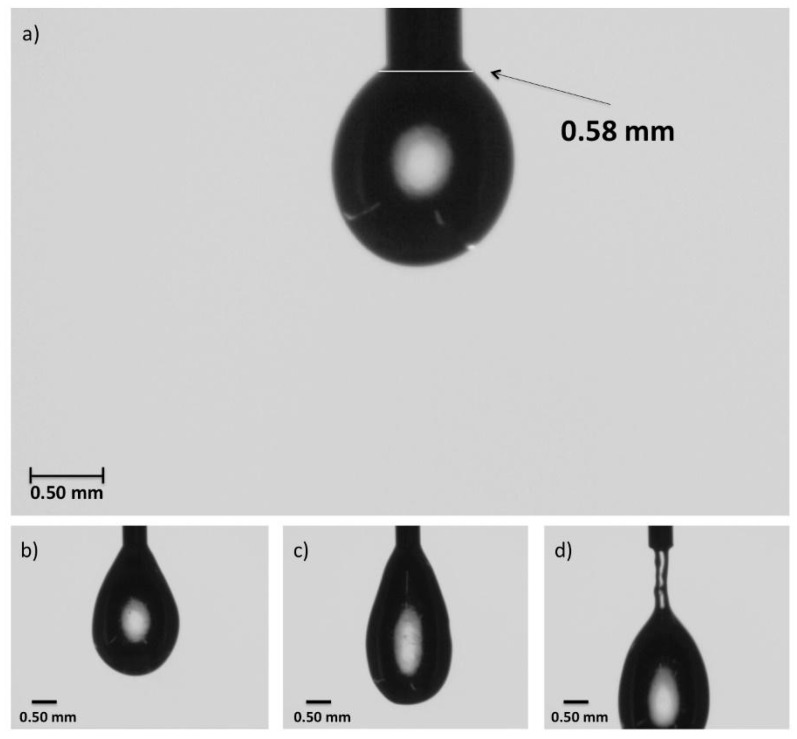
Die swell test image sequence with HG-6 in a Nordson precision tip with an inner diameter of 0.25 mm: (**a**) the width of the drop was taken at 0.08 mm of the tip, which is the approximate distance the tip is from the platform in the 3D printer; (**b**) growth of drop; (**c**) before separation of the drop from the tip; (**d**) behavior as the drop falls from the tip.

The images taken in the high-speed video camera show the swelling of polymer as it exits the die. This phenomenon was studied for all of the solutions (HG-1 to HG-6), and all resulted in the same magnitude of swelling, 0.56 ± 0.04 mm in diameter. In Figure 7, the sequenced photos of HG-6 taken while a drop of the hydrogel pre-crosslinked solution exits the precision tip can be observed. The width of the hydrogel solution drop was measured 0.08 mm from the end of the tip, which is the distance between the extrusion tip and the build surface in the printing process. With a Nordson tip with an inner diameter of 0.25 mm and the systems of hydrogels used in this work, the best resolution that can be achieved is about 0.60 mm, which translates into an extruded line width of 132% the size of the inner diameter of the tip. 

### 3.6. Extrusion Test and Printing

**Figure 8:**
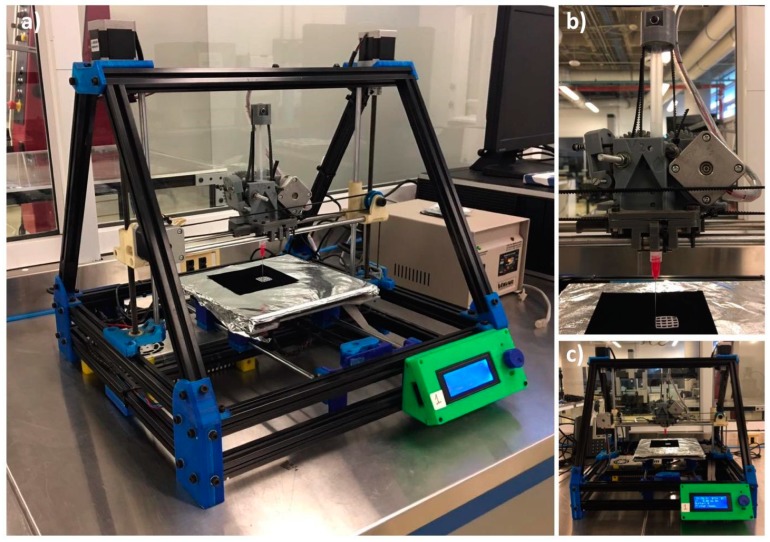
RepRap Mendelmax 3D printer with the modified extruder for the hydrogel printing: (**a**) Isometric view; (**b**) Close-up view from the modified extruder; (**c**) Front view.

For the extrusion test, a modified RepRap Mendelmax 3D printer (Figure 8) was used. The G-code was generated with the software Cura. The designs shown in Figure 1 were printed. After printing the design in the build platform, the structure was submerged in a 6 *w*/*v* % solution of sodium chloride for a complete crosslinking of the alginate. 

**Figure 9:**
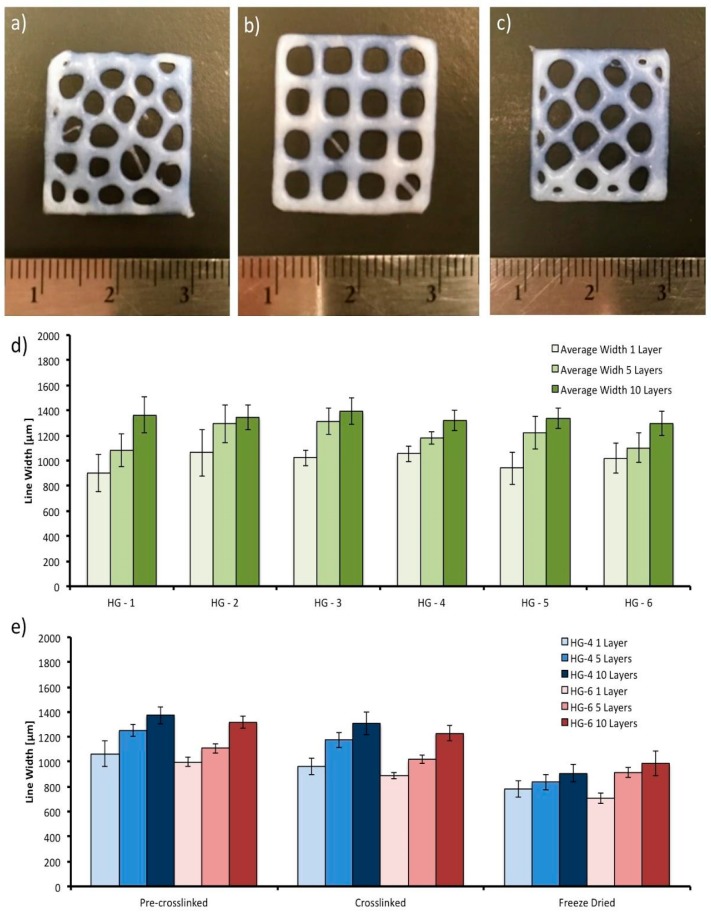
Printed hydrogel designs. From left to right and top to bottom: (**a**) voronoi printed design; (**b**) grid printed design; (**c**) hexagon printed design; (**d**) average line width of the printed hydrogel systems (HG-1 to HG-6) in the grid design with one, five, and ten layers; (**e**) average line width of the hydrogel in the different processes, after printing (pre-crosslinked), after crosslinking, and after freeze drying.

In Figure 9a–c, the three different printed designs (grid, hexagon, and Voronoi) can be observed. Following He et al. recommendations, the space between the lines of the grid increased to reduce fusion and diffusion of the lines and layers. The images presented in Figure 9a,c were captured after the deposition process of the pre-crosslinked solutions on the platform. The phenomenon of hydrogel diffusion (dumbbell form) [41] can be seen. It appeared on the intersections of the lines of the structures due to the gravity. Fusion, another phenomenon due to gravity, which is the main reason for the lack of resolution in the printed designs, is seen on the structures where the weight of the top layers deform the layers that are below. Overlap on the corners is clearly seen on the hexagon and voronoi structures (Figure 9a,c), which is a product of the double extrusion on the same area, where the angle of separation of the two contiguous lines is less than 45°. Quantitative results were obtained for the layer deformation with the grid design (one, five, and ten layers; 0.15, 0.75, and 1.5 mm of height, respectively). The results showed no statistical difference in performance between the six different hydrogel systems, as can be observed from the graph in Figure 9d. The behavior of the systems is consistent with the results of the viscoelasticity of the hydrogels, as there is also no statistical difference between them with the addition of different factors in their composition. However, there was an increase in the line width of the design with one layer (0.90 mm average line width), compared to the design with five layers (1.15 mm average line width), and subsequently in the design with ten layers (1.30 mm average line width), following the fusion behavior mentioned previously with the weight of the top layers acting upon the bottom layers.

The processes of the printed design—crosslinking and freeze drying—can also affect the final measurements of the sample. The line widths of the printed grid design in the two hydrogel systems that presented the best mechanical properties (HG-4 and HG-6) were measured after printing (pre-crosslinked hydrogel), after crosslinking in the calcium chloride solution, and finally after freeze drying. As can be observed from the graph in Figure 9e, there was an average line width reduction of 7.8% after crosslinking, and an average total reduction of 27.5% after freeze drying. The reduction in size after crosslinking is caused by the ionic bonding between the excess of Ca^2+^ used and the alginate chains, which come closer to each other after the chemical reaction. Further reduction in the freeze-drying process is caused by the fact that free volume is reduced when the water molecules are removed from the structure and given the low strength of the ionic bonding of the alginate, shrinkage between the crosslinked biopolymer chains happen.

## 4. Conclusions

In this study, alginate/gelatin composite hydrogel pre-crosslinked solutions were prepared, and their 3D-printability was studied. First, the effect of pre-crosslinking percentage was investigated, where formulations with greater storage modulus at almost no shear conditions were identified and proved better for 3D printing. With the characterization of the crosslinked hydrogels, it was determined that the compositions used in this research showed an adequate average pore size from 150 to 200 μm for tissue regeneration. Furthermore, tensile tests showed that the hydrogels stand in the lower range of the ideal elastic modulus, as observed in some oral soft tissues. The highest elastic modulus was accomplished with the alginate/gelatin composite hydrogel containing TiO_2_ (HG-4), 20 MPa. It was observed that even though the addition of gelatin or nanoparticles did not affect the pre-crosslinked solution viscoelastic properties, it did affect its mechanical properties after crosslinking of the alginate. Finally, in the extrusion test and printing, with the parameters described in the experimental design, it was proved that various designs with multiple layers of 0.15 mm could be printed. Designs with a line-structure will lose resolution as the height is increased due to deformation of the bottom layers of the design due to the increasing weight of the top layers. However, there is a reduction of the width and height of the structure after it is crosslinked (7.8%) and freeze-dried (27.5%) compared to the initial printed design. Addition of gelatin, TiO_2_, or β-TCP to the pre-crosslinked alginate solution does not affect printability. The study provides new insights into the possibilities of alginate/gelatin added with TiO_2_ nanoparticles and β-TCP composite hydrogel 3D printing for tissue regeneration, where further assays are being made to prove cytotoxicity, cell proliferation, and bioactivity of the material.

## Figures and Tables

**Table 1:** Summary of the compositions of the hydrogels in weight/volume percentage.

*Hydrogel*	ALG [*w*/*v* %]	GEL [*w*/*v* %]	TiO_2_ [*w*/*v* %]	TCP [*w*/*v* %]	CaCl_2_ [*w*/*v* %]
*HG-1*	2.0	-	-	-	0.2
*HG-2*	2.0	0.5	-	-	0.2
*HG-3*	2.0	-	0.1	-	0.2
*HG-4*	2.0	0.5	0.1	-	0.2
*HG-5*	2.0	0.5	-	1.0	0.2
*HG-6*	2.0	0.5	0.1	1.0	0.2

## References

[1] Jensen S.S., Terheyden H. (2009). Bone augmentation procedures in localized defects in the alveolar ridge: Clinical results with different bone grafts and bone-substitute materials. Int. J. Oral Maxillofac. Implants.

[2] Silva F.M.S., Cortez A.L.V., Moreira R.W.F., Mazzonetto R. (2006). Complications of intraoral donor site for bone grafting prior to implant placement. Implant Dent..

[3] Holtzclaw D., Toscano N., Eisenlohr L., Callan D. (2008). The Safety of Bone Allografts Used in Dentistry: A Review. J. Am. Dent. Assoc..

[4] Fisher J.P., Reddi A.H. (2003). Functional tissue engineering of bone: Signals and scaffolds. Top. Tissue Eng..

[5] Zhu J., Marchant R.E. (2011). Design properties of hydrogel tissue-engineering scaffolds. Expert Rev. Med. Devices.

[6] Ahmed E.M. (2015). Hydrogel: Preparation, characterization, and applications: A review. J. Advert. Res..

[7] El-Sherbiny I.M., Yacoub M.H. (2013). Hydrogel scaffolds for tissue engineering: Progress and challenges. Glob. Cardiol. Sci. Pract..

[8] Murphy C.M., O’Brien F.J. (2010). Understanding the effect of mean pore size on cell activity in collagen-glycosaminoglycan scaffolds. Cell Adhes. Migr..

[9] Maiti B., Díaz Díaz D. (2018). 3D Printed Polymeric Hydrogels for Nerve Regeneration. Polymers.

[10] Josef E., Zilberman M., Bianco-Peled H. (2010). Composite alginate hydrogels: An innovative approach for the controlled release of hydrophobic drugs. Acta Biomater..

[11] Webber R.E., Shull K.R. (2004). Strain Dependence of the Viscoelastic Properties of Alginate Hydrogels. Macromolecules.

[12] Sarker B., Singh R., Silva R., Roether J.A., Kaschta J., Detsch R., Schubert D.W., Cicha I., Boccaccini A.R. (2014). Evaluation of fibroblasts adhesion and proliferation on alginate-gelatin crosslinked hydrogel. PLoS ONE.

[13] Contessi Negrini N., Bonnetier M., Giatsidis G., Orgill D.P., Farè S., Marelli B. (2019). Tissue-mimicking gelatin scaffolds by alginate sacrificial templates for adipose tissue engineering. Acta Biomater..

[14] You F., Wu X., Chen X. (2017). 3D printing of porous alginate/gelatin hydrogel scaffolds and their mechanical property characterization. Int. J. Polym. Mater. Polym. Biomater..

[15] Gnanaprakasam Thankam F., Muthu J. (2014). Alginate based hybrid copolymer hydrogels—Influence of pore morphology on cell–material interaction. Carbohydr. Polym..

[16] Liu Q., Li Q., Xu S., Zheng Q., Cao X. (2018). Preparation and Properties of 3D Printed Alginate–Chitosan Polyion Complex Hydrogels for Tissue Engineering. Polymers.

[17] Kühn P., Rozenbaum R., Perrels E., Sharma P., van Rijn P. (2017). Anti-Microbial Biopolymer Hydrogel Scaffolds for Stem Cell Encapsulation. Polymers.

[18] Luo Y., Lode A., Akkineni A.R., Gelinsky M. (2015). Concentrated gelatin/alginate composites for fabrication of predesigned scaffolds with a favorable cell response by 3D plotting. RSC Adv..

[19] Giannitelli S., Mozetic P., Trombetta M., Rainer A. (2015). Additive Manufacturing of Pluronic/Alginate Composite Thermogels for Drug and Cell Delivery. Additive Manufacturing.

[20] Izadifar Z., Chang T., Kulyk W., Chen X., Eames B.F. (2016). Analyzing Biological Performance of 3D-Printed, Cell-Impregnated Hybrid Constructs for Cartilage Tissue Engineering. Tissue Eng. Part C Methods.

[21] Bendtsen S.T., Quinnell S.P., Wei M. (2017). Development of a novel alginate-polyvinyl alcohol-hydroxyapatite hydrogel for 3D bioprinting bone tissue engineered scaffolds. J. Biomed. Mater. Res. A.

[22] Barradas A.M.C., Yuan H., van Blitterswijk C.A., Habibovic P. (2011). Osteoinductive biomaterials: Current knowledge of properties, experimental models and biological mechanisms. Eur. Cell. Mater..

[23] Zazakowny K., Lewandowska-Łańcucka J., Mastalska-Popławska J., Kamiński K., Kusior A., Radecka M., Nowakowska M. (2016). Biopolymeric hydrogels—Nanostructured TiO_2_ hybrid materials as potential injectable scaffolds for bone regeneration. Colloids Surf. B Biointerfaces.

[24] Kim S.-Y., Bark C.W., Van Quy H., Seo S.-J., Lim J.-H., Lee J.-M., Suh J.-Y., Lee Y., Um H.-S., Kim Y.-G. (2018). Photofunctionalizing effects of hydroxyapatite combined with TiO_2_ on bone regeneration in rabbit calvarial defects. J. Biomed. Mater. Res. B Appl. Biomater..

[25] Tiainen H., Wohlfahrt J.C., Verket A., Lyngstadaas S.P., Haugen H.J. (2012). Bone formation in TiO_2_ bone scaffolds in extraction sockets of minipigs. Acta Biomater..

[26] Shirai R., Miura T., Yoshida A., Yoshino F., Ito T., Yoshinari M., Yajima Y. (2016). Antimicrobial effect of titanium dioxide after ultraviolet irradiation against periodontal pathogen. Dent. Mater. J..

[27] Chen J.-P., Cheng T.-H. (2008). Bone regeneration using 3D hyaluronic acid and chitosan-containing thermo-reversible hydrogel and canine mesenchymal stem cells. J. Biotechnol..

[28] Jose R.R., Rodriguez M.J., Dixon T.A., Omenetto F., Kaplan D.L. (2016). Evolution of Bioinks and Additive Manufacturing Technologies for 3D Bioprinting. ACS Biomater. Sci. Eng..

[29] Yanagawa F., Sugiura S., Kanamori T. (2016). Hydrogel microfabrication technology toward three dimensional tissue engineering. Regen. Ther..

[30] Day M. (2009). Rhino Grasshopper, AEC Magazine.

[31] Malda J., Visser J., Melchels F.P., Jüngst T., Hennink W.E., Dhert W.J.A., Groll J., Hutmacher D.W. (2013). 25th anniversary article: Engineering hydrogels for biofabrication. Adv. Mater..

[32] Guvendiren M., Lu H.D., Burdick J.A. (2011). Shear-thinning hydrogels for biomedical applications. Soft Matter.

[33] Schuurman W., Levett P.A., Pot M.W., van Weeren P.R., Dhert W.J.A., Hutmacher D.W., Melchels F.P.W., Klein T.J., Malda J. (2013). Gelatin-methacrylamide hydrogels as potential biomaterials for fabrication of tissue-engineered cartilage constructs. Macromol. Biosci..

[34] Aguado B.A., Mulyasasmita W., Su J., Lampe K.J., Heilshorn S.C. (2012). Improving viability of stem cells during syringe needle flow through the design of hydrogel cell carriers. Tissue Eng. Part A.

[35] Macosko C.W. (1994). Rheology: Principles, Measurements, and Applications.

[36] Das D., Bang S., Zhang S., Noh I. (2017). Bioactive Molecules Release and Cellular Responses of Alginate-Tricalcium Phosphate Particles Hybrid Gel. Nanomaterials.

[37] Zhao K., Feng L., Li Z., Fu Y., Zhang X., Wei J., Wei S. (2014). Preparation, characterization and photocatalytic degradation properties of a TiO_2_/calcium alginate composite film and the recovery of TiO_2_ nanoparticles. RSC Adv..

[38] Dong Z., Wang Q., Du Y. (2006). Alginate/gelatin blend films and their properties for drug controlled release. J. Membr. Sci..

[39] McKee C.T., Last J.A., Russell P., Murphy C.J. (2011). Indentation versus tensile measurements of Young’s modulus for soft biological tissues. Tissue Eng. Part B Rev..

[40] Goktas S., Dmytryk J.J., McFetridge P.S. (2011). Biomechanical behavior of oral soft tissues. J. Periodontol..

[41] He Y., Yang F., Zhao H., Gao Q., Xia B., Fu J. (2016). Research on the printability of hydrogels in 3D bioprinting. Sci. Rep..

